# Endoscopic Reduction of Organoaxial Gastric Volvulus Within a Giant Type III Paraesophageal Hernia

**DOI:** 10.14309/crj.0000000000002334

**Published:** 2026-09-28

**Authors:** Rayhan Karimi, Lee Sigmon, Ruchi Shrestha

**Affiliations:** 1 Atrium Health, Charlotte, NC

**Keywords:** gastric volvulus, organoaxial volvulus, paraesophageal hernia, endoscopic reduction, upper gastrointestinal obstruction

## CASE REPORT

A 68-year-old woman with a history of hiatal hernia presented with acute-onset severe epigastric pain, nausea, and multiple episodes of vomiting. Computed tomography demonstrated a large hiatal hernia with marked gastric distention and probable gastric outlet obstruction (Figure 1). A nasogastric tube was placed, and she was transferred for endoscopic management. Esophagogastroduodenoscopy revealed a large type III paraesophageal hernia containing the gastroesophageal junction and stomach with retained food and liquid debris (Figure 2). A point of torsion between the gastric body and antrum established the diagnosis of organoaxial gastric volvulus (Figure 3). After gastric decompression, the scope was carefully advanced across the torsion and then gradually withdrawn with gentle torque opposite the direction of rotation, successfully reducing the volvulus (Figure 4). An endoscopically placed nasogastric tube was left for continued decompression. Subsequent upper gastrointestinal contrast study confirmed restoration of gastric emptying, and the patient later underwent definitive laparoscopic paraesophageal hernia repair. Gastric volvulus is an uncommon but potentially life-threatening complication of large paraesophageal hernias that requires prompt recognition and intervention.^1–3^ This case highlights the impressive size of the hiatal hernia on cross-sectional imaging and the rare endoscopic visualization of gastric volvulus before and after reduction, illustrating the important diagnostic and therapeutic role of esophagogastroduodenoscopy in the management of gastric volvulus.

**Figure 1.**
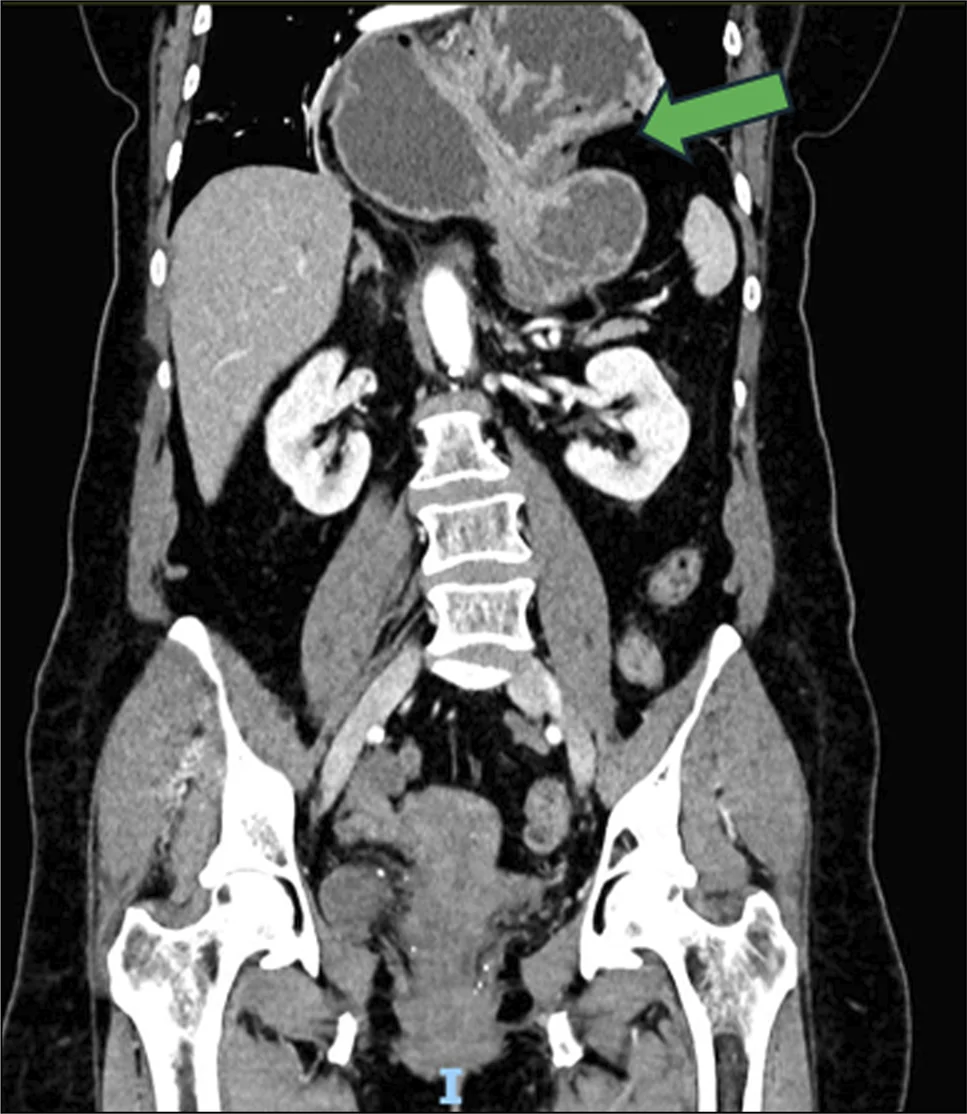
Coronal contrast-enhanced computed tomography demonstrating a giant type III paraesophageal hernia with marked gastric distention, focal constriction at the diaphragmatic hiatus, and herniation of the distal stomach into the thorax (green arrow).

**Figure 2.**
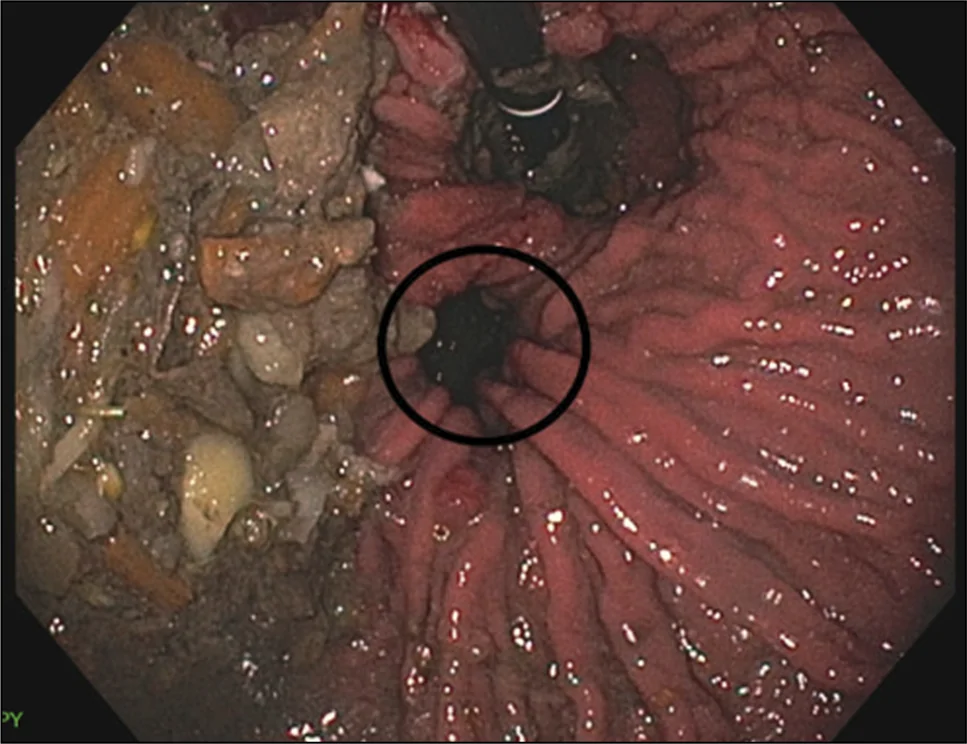
Retroflexed endoscopic view demonstrating friable gastric mucosa with retained food debris and the herniated gastric lumen (circled).

**Figure 3.**
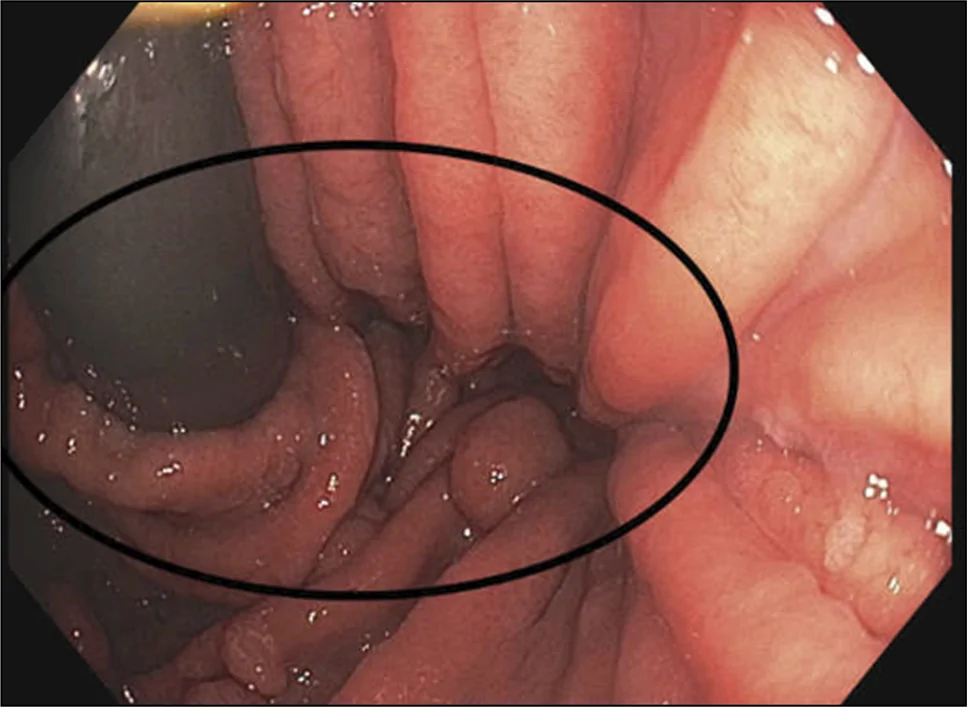
Retroflexed view demonstrating the gastric volvulus at the point of torsion (circled).

**Figure 4.**
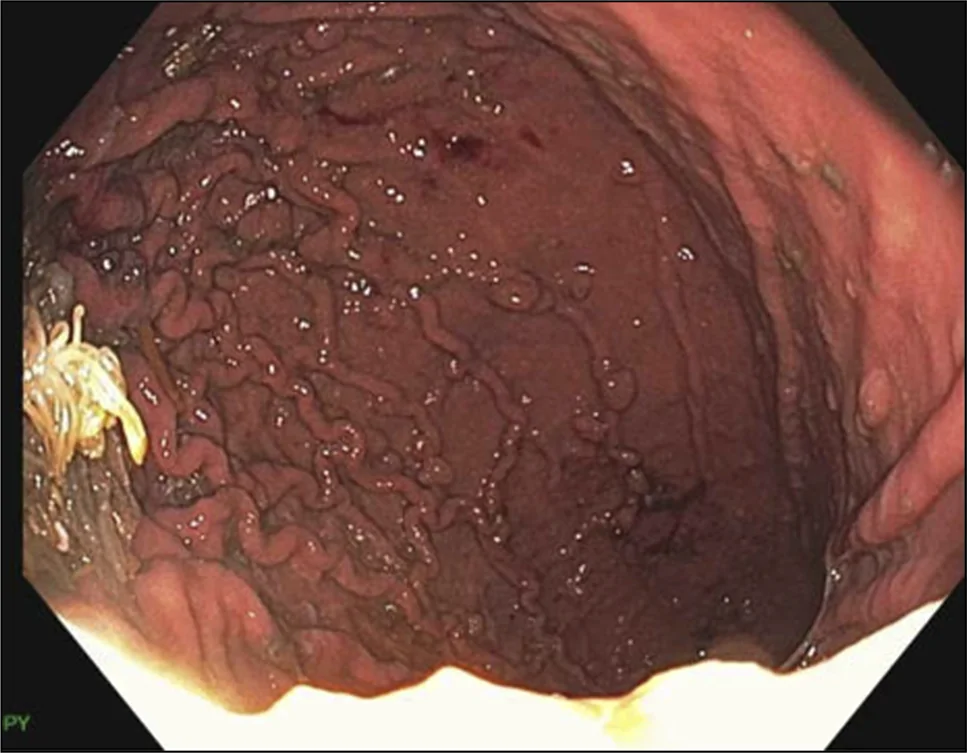
Direct endoscopic view of the stomach following detorsion, suction, and decompression demonstrating restoration of normal gastric anatomy.

## DISCLOSURES

Author contributions: All authors had substantial contributions to conception of work, drafting the work, and accountability for all aspects of the work. R. Karimi is the article guarantor.

Financial disclosure: None to report.

Informed consent was obtained for this case report.
